# Airborne Microorganisms at Hellenic Atmospheric Aerosol and Climate Change Station in Helmos Mountain (Greece)

**DOI:** 10.1021/acsearthspacechem.5c00064

**Published:** 2025-06-12

**Authors:** Eleftheria Katsivela, Sofia Eirini Chatoutsidou, Aggeliki Saridaki, Louiza Raisi, Panagiota Stathopoulou, Georgios Tsiamis, Gao Kunfeng, Prodromos Fetfatzis, Foskinis Romanos, Marilena Gidarakou, Maria I. Gini, Konstantinos Granakis, Maria Mylonaki, Christina Papanikolaou, Stergios Vratolis, Francisca Vogel, Olga Zografou, Ottmar Möhler, Alexandros Papayannis, Konstantinos Eleftheriadis, Athanasios Nenes, Mihalis Lazaridis

**Affiliations:** † Department of Electronic Engineering, Hellenic Mediterranean University, 3 Romanou st., Chania 73133, Greece; ‡ Atmospheric Aerosols Laboratory, School of Chemical and Environmental Engineering, Technical University of Crete, University Campus, Chania 73100, Greece; § Laboratory of Systems Microbiology and Applied Genomics, Department of Sustainable Agriculture, University of Patras, 2 G. Seferi st., Agrinio 30100, Greece; ∥ Laboratory of Atmospheric Processes and their Impacts, School of Architecture, Civil & Environmental Engineering, Ecole Polytechnique Fédérale de Lausanne, Lausanne CH-1015, Switzerland; ⊥ Environmental Radioactivity & Aerosol technology for atmospheric and Climate impacT Lab, INRaSTES, N.C.S.R. “Demokritos”, Ag. Paraskevi, Athens 15310, Greece; # Center for the Study of Air Quality and Climate Change, Institute of Chemical Engineering Sciences, Foundation for Research and Technology Hellas, Patras 265 04, Greece; ∇ Laser Remote Sensing Unit, Department of Physics, National and Technical University of Athens, Zografou 15780, Greece; ○ Meteorological Institute, Ludwig-Maximilians-Universität München, Munich 80539, Germany; ◆ National Research Council of Italy - Institute of Methodologies for Environmental Analysis, Potenza 85050, Italy; ¶ Institute of Meteorology and Climate Research, Karlsruhe Institute of Technology (KIT), Karlsruhe 76131, Germany

**Keywords:** airborne microorganisms, bacterial diversity, fluorescent particles, aerosols, ice nuclei, Saharan dust

## Abstract

Cultural-based methods of bacteria and fungi and molecular identification of bacteria were combined with parallel continuous measurements of aerosol chemical composition, number size distribution, ice-nucleating concentration, and fluorescent particle size distribution and characteristics. Measurements took place at the Helmos Hellenic Atmospheric Aerosol and Climate Change Station (HAC)^2^, Greece, during the 2021 CALISHTO campaign. The objective was to characterize the microorganism levels at the (HAC)^2^ station and further investigate the associations between bioaerosols and aerosols in atmospheric processes that play a key role in the formation of ice crystals. Very low concentrations of viable, cultivable heterotrophic bacteria (4 ± 4 CFU/m^3^) were measured, whereas fast-growing fungi were not affected (182 ± 86 CFU/m^3^) by the environmental conditions at the station. The size distribution of heterotrophic bacteria was bimodal with peaks at fine (1.1–2.1 μm) and coarse size fractions (*d* > 7 μm), whereas airborne fungi exhibited a monomodal distribution (2.1–3.3 μm). Bacterial populations identified using 16S rRNA correlated well (*r* = 0.82) with the averaged concentrations of fluorescent particles (A and C channels). Strong correlations were obtained between total bacterial and particle volume concentrations of coarser fractions (>1 μm, 0.61–0.86), suggesting their strong presence in these sizes. No correlation was found with ice nuclei (INP) (*r* = −0.04) and low to medium negative correlations with the organics and ions (SO_4_^2–^, NH_4_^+^, NO_3_^–^, Cl^–^) possibly due to their relatively lower sizes. In accordance with the culture-dependent analysis, relatively low total bacterial concentrations were determined by real-time PCR, with concentrations ranging from 33.4 to 117.2 GE/m^3^. High bacterial diversity was found with 123 bacterial Operational Taxonomy Units (OTUs) classified in 10 phyla, 16 classes, 56 families, and 78 genera. Origin of the air masses was a significant driver to bacterial communities. Enrichment of specific species such as and was observed during Saharan dust episodes, while in the presence of continental air masses, characteristic species such as *Rhizobium sp*., *Corynebacterium sp*., and had higher relative abundance. Our study provides a comprehensive analysis and quantification of the varying drivers and variability in microorganisms in high-altitude site.

## Graphical abstract

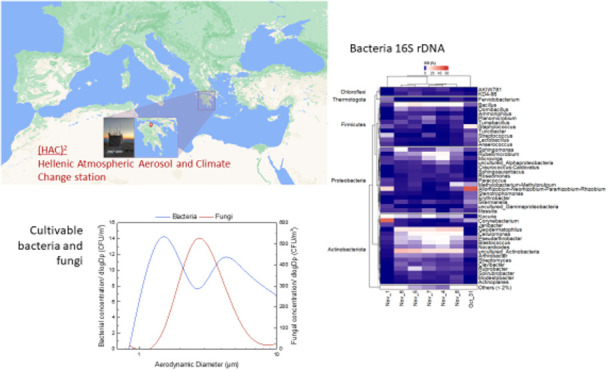

## 1 Introduction

Biological aerosol, or bioaerosol, are ubiquitous in the atmosphere^1^ and are released from both natural sources and anthropogenic activities.^2–6^ Primary bioaerosol sources are soil and vegetation, although considerable sources can be found in oceanic regions with high primary productivity. Perhaps the least understood of all atmospheric aerosol, bioaerosols can affect cloud formation by acting as cloud condensation nuclei (CCN) and ice nuclei (INP),^3,7–15^ thus potentially modulating regional and global climate.^16^ Bioaerosols can be excellent ice nuclei and induce ice formation (hence precipitation) in warm mixed-phase cloud temperatures when other aerosol types, like dust, cannot.^14^ Their cloud glaciation impact can be substantially magnified by ice multiplication processes^11^ that occur in warm mixed-phase cloud temperatures. Reducing the uncertainty in predicting bioaerosol concentrations is important for better predictions of INPs, especially in a postfossil world where anthropogenic sources of INP will be reduced.

The ice nucleation activity (INA) of bacteria has been attributed to an outer membrane-bound protein of around 120–180 kDa in size, which is encoded by a single gene, *inaZ*, proposed to provide a template for the arrangement of water molecules in crystals similar to hexagonal ice. Many of the ice-nucleating bacterial strains such as *Pseudomonas sp*., *Erwinia sp*., and *Xanthomonas sp*. have been isolated from the surface of plants, introducing the phyllosphere as a key player of bioprecipitation cycles. Sands et al.^17^ proposed the “bioprecipitation feedback mechanism”, in which plant-associated microorganisms are transported to the altitude of clouds as aerosols and incite precipitation via their INA, which is in turn is beneficial for ecosystem health as it helps maintain water vapor within the ecosystem (rapid recycling of water), and at the same time limits the time that bacteria remain airborne—offering both organism-level and ecosystem-level advantages. Limited information is also known about CCN activity arising from airborne bacteria. Bauer et al.^18^ confirmed that both Gram-positive and Gram-negative bacteria were activated at supersaturations where wettable particles of the same size would not be activated. Additionally, Sharma and Rao^19–21^ showed that several bacteria exhibited satisfactory wettability (water contact angle <16°) that makes them eligible to act as condensation nuclei. Besides low contact angle, the water-soluble organic fraction is suggested as an important property that influences CCN activity of microorganisms (e.g., 2, 20, 21). Lazaridis^22^ showed that bacteria activation (especially ) in the atmosphere is a favorable process. It is well established that certain bacterial species trigger condensation of water vapor and thus increase the ability to activate cloud droplets. However, the diversity and abundance of airborne bacteria are still poorly understood. Several studies report that the bacterial profile varies considerably depending on geographical, seasonal, and altitude characteristics.^8,9^ In principle, bacteria concentration is lower at higher altitudes, while the type of environment governs the biodiversity of airborne biological material.^14,23^

The focus of this study was to characterize the airborne microbial community at a typical free tropospheric background site, often with low influence from the surface polluted layers^13,14,24,25^ during the 2021 CALISHTO campaign, using samples collected at the Helmos Hellenic Atmospheric Aerosol and Climate Change Station (HAC)^2^ at Mount Helmos in Peloponnese, Greece. The aim was to characterize the biological component of different air masses originating from continental and Saharan regions at a site that is representative of the Eastern Mediterranean, where very little genomic data is available. In addition, the size distribution characteristics of cultivable heterotrophic bacteria and fast-growing fungi were measured. The influence of Saharan dust episodes on the bacterial community was also studied in comparison to continental microbial populations as well as the correlation between bacteria concentration with averaged concentrations of fluorescent particles, INP levels, and particle volume concentrations. The influence of the Saharan dust episodes was examined by using lidar data and back trajectory analysis.

## 2 Materials and Methods

### 2.1 Helmos Mt (HAC)^2^ Description

The Helmos Atmospheric Aerosol and Climate Change Station (HAC)^2^ is located on mount Helmos (Aroania) in the Northern Peloponnese at an altitude of 2,314 m (22° 20′ East, 37° 98′ North) (Figure 1). The station is the only permanent, high-altitude facility for atmospheric research in the Eastern Mediterranean region. It has the lowest ABL-TopoIndex in Europe, which means that it is an excellent location for Free Troposphere (FT) sampling, though it is partially influenced by the atmospheric boundary layer (ABL) close to the ground, during summer/Fall and especially during midday^13,14,24^ which could provide bioaerosols from the alpine forest below with a strong diurnal cycle.^25^ Additionally, the station lies at the crossroads of air masses from different origins, including continental, Saharan, and long-range biomass burning,^14,26^ allowing the study of ambient aerosol with remarkably different properties. Station data are reported at the World Meteorological Organization (WMO), the Global Atmospheric Watch (GAW) network, and the Aerosol, Clouds and Trace Gases Research Infrastructure (ACTRIS) (actris.eu), under the acronym HAC. (HAC)^2^ is also part of the PANhellenic infrastructure for Atmospheric Composition and climatE chAnge (PANACEA). In the current work, parallel to the microbial sampling, a number of in situ measurements were also performed using different instruments under the international Cloud-AerosoL InteractionS in the Helmos background TropOsphere (CALISTHO) Campaign (https://calishto.panacea-ri.gr/) from September 2021 to March 2022 having the objective of studying the cloud microphysical properties.^13,14,26^

**Figure 1:**
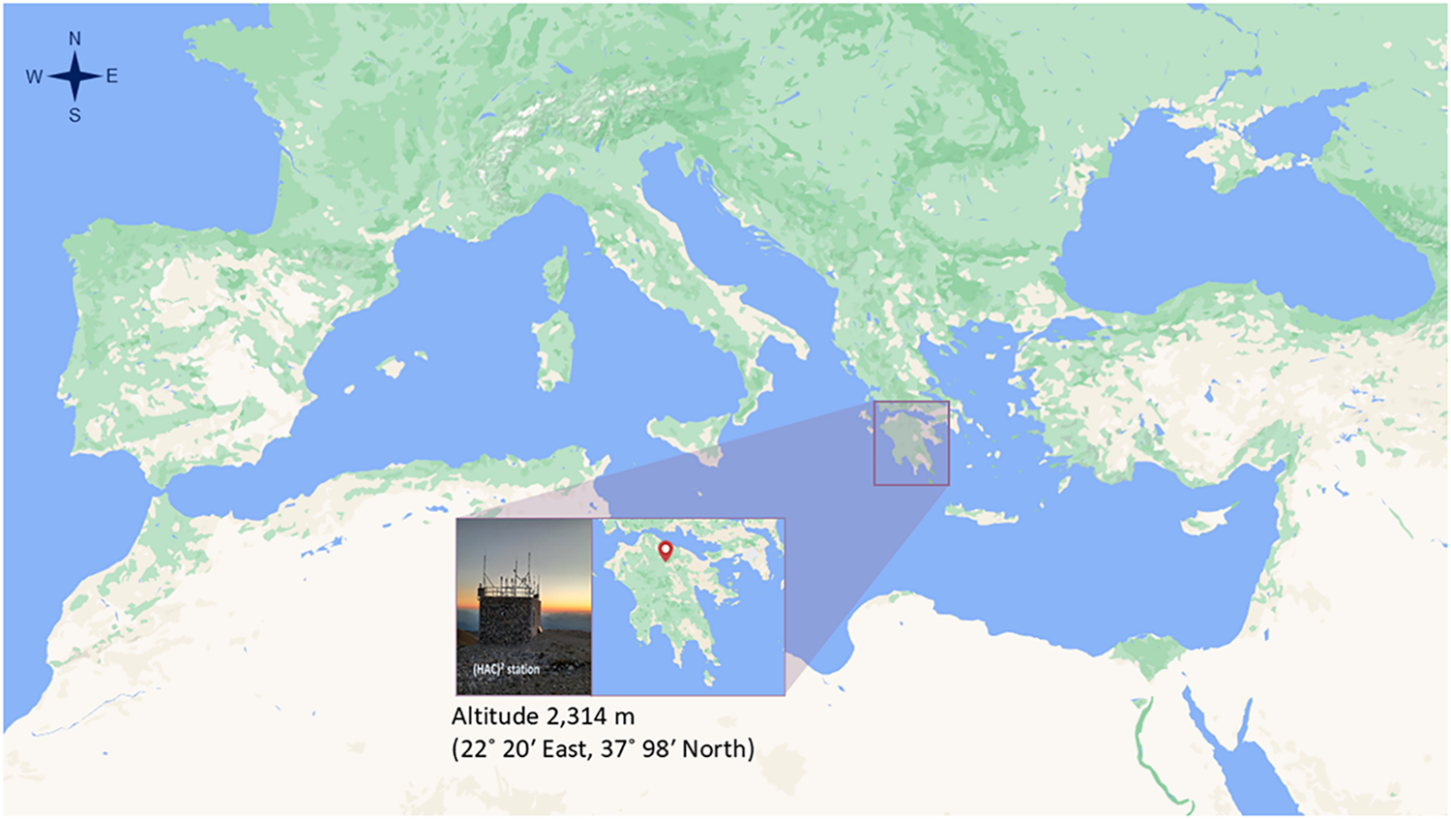
Map of the Mediterranean region and location of the Helmos Hellenic Atmospheric Aerosol and Climate Change (HAC)^2^ Station. (https://mapstyle.withgoogle.com/).

### 2.2 Airborne Microorganisms Sampling and Analysis

#### 2.2.1 Sampling of Viable, Cultivable, Airborne Microorganisms

The viable, cultivable, airborne microorganisms (heterotrophic bacteria and fast-growing fungi) were collected by impaction using a MAS-100 NT sampler (MBV AG, Switzerland) with adapted sampling heads equipped with holes of 300 × 0.6 mm, while their size distribution was measured using six stages (0.65–1.1, 1.1–2.1, 2.1–3.3, 3.3–4.7, 4.7–7, >7 μm), viable Andersen Cascade Impactor (ACI, Thermo Electron Corporation). Sampling took place from 11 a.m. to 3 p.m. on four consecutive days (19–22/10/2021; Table 1). Air volumes of the collected samples, using either the MAS-100 NT sampler or the ACI, were optimized prior to the experiments, so that, for reliable results, the colony number per plate did not exceed 80.^4,27^ Each aluminum orifice stage was disinfected by using wipes containing 70% isopropyl alcohol between collections of different samples. Concentrations of airborne cultivable microorganisms were expressed as colony-forming units per cubic meter of air (CFU m^–3^).

**Table 1:** Sampling Dates, Numbers (*n*), and Instruments Used for the Collection of Airborne Microorganisms

	culture-dependent analysis		molecular analysis	
date	MAS-100 NT^a^ (*n*)	ACI^b^ (*n*)	date	Leckel^c^ (*n*)
19–10–21	1 (*n* 6)	1 (*n* 1)	31–10–21	1 (*n* 1)
20–10–21	1 (*n* 6)	1 (*n* 1)	01–11–21	1 (*n* 1)
21–10–21	1 (*n* 9)	1 (*n* 1)	04–11–21	1 (*n* 1)
22–10–21	1 (*n* 4)	1 (*n* 1)	05–11–21	1 (*n* 1)
			06–11–21	1 (*n* 1)
			07–11–21	1 (*n* 1)
			08–11–21	1 (*n* 1)
*N* total	4 (*n* 25)	4 (*n* 4)	*N* total	7 (*n* 7)

a MAS-100 NT sampler (MBV AG, Switzerland).

b Viable Andersen Cascade Impactor (ACI, Thermo Electron Corporation).

c Leckel air sampler LVS6-RV (Leckel, Germany).

#### 2.2.2 Microbial Cultivation

Air sampled microorganisms were cultivated in 90 mm Petri dishes containing specific microbiological growth media.^4,27^ The heterotrophic bacteria were cultivated in Tryptone Soy Broth (Merck, Germany) containing 1.5% (w/v) agar at 37 °C in the dark for 48 h. An incubation temperature of 37 °C was chosen for the determination of the airborne (opportunistic pathogenic) heterotrophic bacteria. The viable, fast-growing fungi were cultivated in Malt Extract Broth (Lab M, England) containing 1.5% (w/v) agar at 20 °C in the dark for 72 h. The time required for the growth of the fast-growing fungi is short compared to other fungi which grow in 5 days or longer at temperatures close to 25 °C. Fast-growing fungi were determined as a representative fraction of airborne cultivable fungi which were also determined at lower temperatures. The counted number of colonies was precisely corrected by using the positive hole conversion tables supplied by the sampler manufacturer.

#### 2.2.3 Sampling of Total Airborne Bacteria and DNA Extraction

Airborne bacteria were collected using a low-volume air sampler Leckel LVS6-RV (Leckel, Germany) equipped with a prehead of PM_10_, operating at 2.3 m^3^/h for 6 h (from 10 a.m. to 4 p.m.). Sampling took place between 31/10/2021 and 1/11/2021 and between 4/11/2021 and 8/11/2021. Microbial DNA was collected on sterilized 47 mm PTFE TE 36 filters with 0.45 μm pore diameter (Whatman). Upon sampling, filters were placed in sterile Petri dishes sealed with parafilm and stored at −20 °C until further processing. Table 1 summarizes the sampling dates and instruments used for the collection of airborne microorganisms.

DNA was extracted from filters using a DNeasy PowerSoil Pro kit (Qiagen). Filters emerged into the lysis buffer of the bead beating tube and were heated to 60 °C for 15 min followed by 15 min of vortex shaking. The remaining steps of DNA extraction were performed according to the manufacturer’s instructions. The purified DNA was stored at −20 °C for subsequent amplification by polymerase chain reaction (PCR) and Illumina MiSeq sequencing. In order to verify that the microorganisms detected originated from the air microbiome under investigation, sterile filters were subjected to DNA extraction in parallel with the rest of the study samples. No amplification product from the blank filter pieces was detected in subsequent stages.

#### 2.2.4 Real-Time Polymerase Chain Reaction (PCR) Amplification and Quantification

Using the universal bacterial primers and probe set outlined in Nadkarni et al.,^28^ total bacterial DNA was measured by real-time PCR (qPCR), as previously reported by Chatoutsidou et al.^29^ and Saridaki et al.^30^ The CFX Connect Real-Time PCR Detection System (Biorad) was utilized to prepare the reactions. Under the following conditions, amplification was performed in 20 μL reactions using 1 μL of template, 10 μL of 2x KAPA Probe Fast Universal qPCR Master Mix (Roche), and 0.2 μM final concentrations of each primer and the dual-labeled probe: 3 min at 95 °C, 40 cycles of 15 s at 95 °C, and 1 min at 60 °C. Every run contained no-template controls with molecular grade water (control), and each sample was measured three times. Every sample’s cycle threshold was compared to a standard curve, and the outcome was given as a sample’s target genome count. Using triplicates of serial dilutions of () DSM498 strain DNA, which had previously been quantified using the Qubit dsDNA High Sensitivity Assay with Qubit 4 Fluorometer (Thermo Fisher Scientific), the standard curve for the DNA quantification was created. The genomes at the standard curve serial dilutions varied from 10^1^ to 10^5^ copies (minimum *R* ^2^ = 0.992). Using the default settings of Biorad CFX Manager software v3.1 (Biorad), real-time PCR running control, baseline correction, cycle threshold (Ct) value determination, and standard curve production were carried out. In order to eliminate between-run variation, factor-qPCR software version 2020.0 was utilized.^31^ The final DNA concentrations were reported as genome equivalents (GE) per cubic meter of air (GE m^–3^).

#### 2.2.5 16S rRNA Region Amplicon Sequencing and Bioinformatic Analysis

16S rRNA amplicon sequencing of airborne bacteria and bioinformatic analysis was performed as described in Chatoutsidou et al.^29^ and Saridaki et al.^30^ Briefly, the hypervariable V3–V4 region of the 16S rRNA gene was amplified using the MiSeq universal primers 341F and 805R^32^ fused to Illumina adapters. Two PCR reactions were performed: the first to amplify the target hypervariable region (460 bp belonging to the V3–V4 region of the bacterial 16S rRNA), and the second to include Illumina index primers. The resulting amplicons were purified using Macherey-Nagel’s NucleoMag NGS Clean-up and Size Selection kit (MACHEREY-NAGEL GmbH & Co, Düren, Germany) according to the manufacturer’s recommendations. Purified samples were suspended in 30 μL of sterile deionized water, and their concentration was measured with a Quawell Q5000 microvolume UV–vis spectrophotometer (Quawell, San Jose, CA). All samples were diluted to a final concentration of 8 nM and mixed equimolarly. The library was sequenced on an Illumina MiSeq sequencing platform by Macrogen (Korea). Sequencing reads were demultiplexed and converted to FASTQ. DNA sequencing data were analyzed using USEARCH^33^ and QIIME.^34^ Paired-end reads were merged, trimmed, and filtered for quality, followed by determining unique sequences and their abundance. Operational taxonomy units (OTUs) were clustered at a 97% similarity threshold using UPARSE algorithm.^35^ Taxonomy was assigned based on BLAST+ algorithm^36^ against SILVA database,^37^ excluding nonbacterial OTUs. The data sets have been deposited to NCBI Sequence Read Archive database under BioProject PRJNA941144.

### 2.3 Aerosol In Situ Measurements

A portable ice nucleation experiment (PINE)^38^ chamber was deployed for automated real-time observations of ice-nucleating particles at (HAC)^2^. In addition, a wideband integrated bioaerosol sensor, New Electronics Option (WIBS-5/NEO, Droplet Measurement Technologies, LLC), was used to record the concentration of aerosol particles in the size range of 0.5 to 30 μm (optical diameter). The WIBS also measures the fluorescent property of aerosol particles on a single particle basis using ultraviolet light to trigger the excitation of particles and then probing the emissions of biofluorophores of the particle.

Furthermore, the chemical composition of nonrefractory species (NRS) of submicron ambient aerosols (PM_1_), including organics (Org), sulfate (SO_4_), nitrate (NO_3_), ammonium (NH_4_), and chloride (Cl) were monitored by a Time-of-Flight Aerosol Chemical Speciation Monitor (ToF-ACSM, Aerodyne Research Inc.), with a time resolution of 10 min. A PM_2.5_ virtual impactor equipped with a Nafion drier was installed in the inlet of the ToF-ACSM. The Relative Ionization Efficiencies (RIEs) for organics, NO_3_– and Cl– were 1.4, 1.1, and 1.3, respectively. The collection efficiency (CE) was derived by comparing the total mass of PM_1_ from a mobility particle size spectrometer (TROPOS-MPSS) (i.e., by summing up the total number of particles across all sizes) with that of the ToF-ACSM plus the equivalent Black Carbon (eBC).^39^ The CE for this campaign was found to be 0.28,^26^ eBC concentrations were obtained from the absorption at 660 nm from the harmonized data set of an AE31 aethalometer (Magee Sci.) and a continuous light absorption photometer (CLAP, NOAA),^40^ which sampled through a PM_10_ cutoff inlet, after correcting for loading and multiscattering effects. For the TROPOS-MPSS (10–800 nm), the number size distribution data were corrected for diffusion losses within the aerosol inlet and the instrument’s internal tubing (using TROPOS-MPSS inversion software) according to the “equivalent length” method.^41^ Additionally, the number size distribution in the size range of 0.3–10 μm was obtained by using an Optical Particle Counter (GRIMM). Also a wind lidar was used to characterize the height of the Planetary Boundary Layer (PBL). All of the aerosol inlets at (HAC)^2^ were equipped with PM_10_ heads and Nafion dryers (RH < 40%).

### 2.4 Lidar

During the CALISHTO Campaign the mobile multiwavelength AIAS lidar system of the National Technical University of Athens (NTUA) in cooperation with the Biomedical Research Foundation Academy of Athens (BRFAA), was used to provide the vertical profiles of the aerosol backscatter coefficient (b_aer_) at 532 and 1064 nm, as well as the particle linear depolarization ratio (δ_pldr_) at 532 nm (spatial resolution of 15 m, every 90 s).^42^ The lower height detection was 250 m above mean sea level (a.m.s.l.). The relative uncertainty in the retrieved b_aer_ and δ_pldr_ profiles was 11 ± 8% and 16 ± 11%, respectively.^43^

### 2.5 Air Mass Origin Modeling

The Flexible Particle Dispersion Model (FLEXPART)^44,45^ was used to determine the residence time of remotely transported aerosol particles in each geographic grid cell (1° × 1°). Backward mode was used for investigating the origin of air masses with respect to microbiome samplings.^14^ The model was applied to simulate the transport of aerosol particles having a geometric mean diameter of 400 nm, density of 2 g cm^–3^, and a standard deviation of 3.3. The model was run every 3 h (from 00:00 to 24:00 LTC) by releasing 40,000 air parcels from (HAC)^2^. FLEXPART considers grid-scale wind (as simple trajectory models do) as well as turbulent wind fluctuations and mesoscale wind fluctuations. It also incorporates drift correction (to prevent accumulation of computational particles released) and density correction (to account for the decrease in air density with height). Wet and dry depositions of aerosol particles were also included in the evaluation of the back trajectories.

## 3 Results and Discussion

### 3.1 Concentrations and Size Distributions of Viable, Cultivable Airborne Microorganisms

The mean concentration of cultivable, potentially pathogenic, heterotrophic bacteria at (HAC)^2^ was exceptionally low and varied between 0 and 12 CFU m^–3^ (Figure 2) with average temperature and relative humidity during sampling being 7.0 ± 2.0 °C and 59 ± 24%, respectively. Compared to a coastal station close to sea level (Akrotiri Monitoring Station, 35°31′48.0″N 24°04′12.0″E, Chania, Crete), where milder meteorological conditions prevailed (mean temperature 22.0 ± 0.8 °C; relative humidity 61 ± 3%) (data not shown) during the same time period, the average concentration of heterotrophic bacteria at (HAC)^2^ was about 24 times lower.^5^ Moreover, the size distribution of heterotrophic bacteria (Figure 3) exhibited no particular differences compared to the corresponding measurements at Akrotiri Monitoring Station.^4,5,27^ Bacterial size distribution was bimodal (Figure 3) with the highest concentrations measured in the fine size fraction at aerodynamic diameters of 1.1–2.1 μm, followed by the coarse fraction with aerodynamic diameters >7 μm.

**Figure 2:**
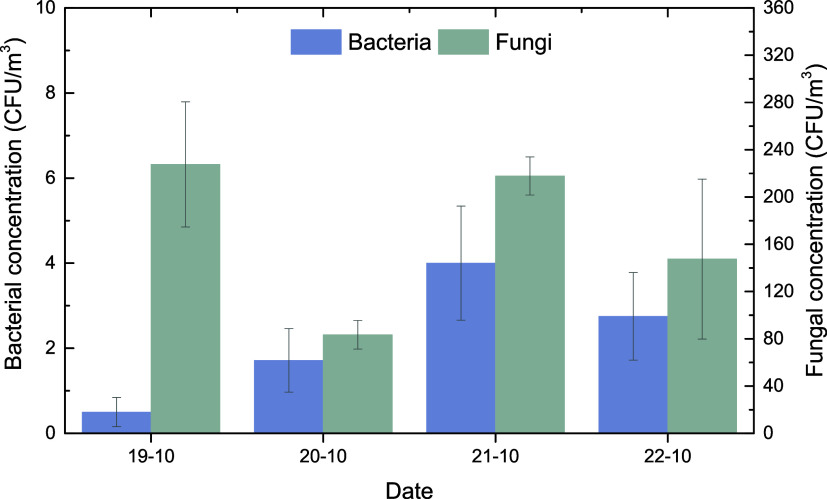
Average (±uncertainty) concentrations for (a) heterotrophic bacteria and (b) fast-growing fungi for each sampling day.

**Figure 3:**
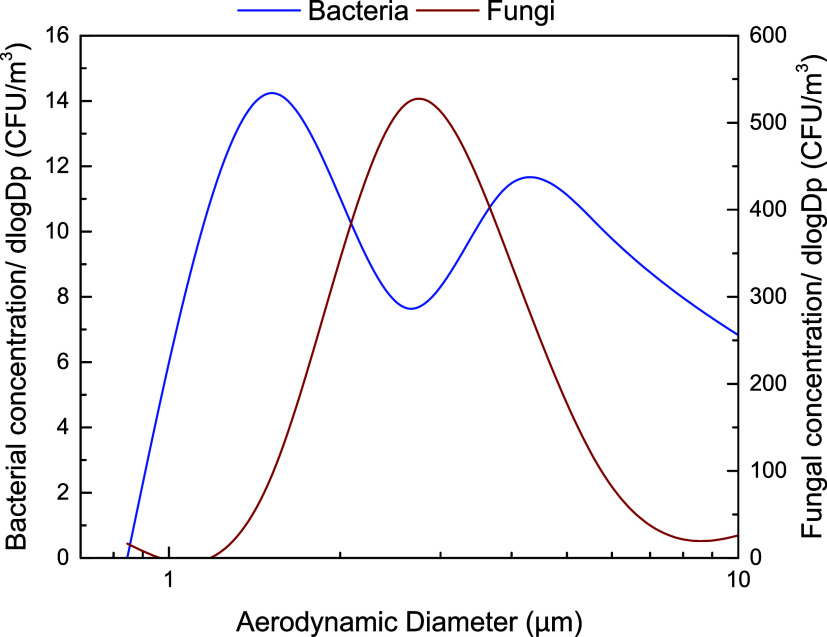
Averaged size distribution of viable, cultivable, airborne heterotrophic bacteria, and fast-growing fungi.

Similarly, to the airborne cultivable bacteria, cultivable, airborne, fast-growing fungi were shown to be less affected by the low temperatures prevailing at the Helmos site as shown in comparison with measurements at the Akrotiri monitoring station at the island of Crete (Greece). Their average concentration was 182 ± 86 CFU m^–3^ (value range 72–314 CFU m^–3^), nearly 3 times lower than the corresponding one at the Akrotiri station. This is in agreement with the study of Haas et al., which showed that no significant differences were detected between the fungal spore concentrations in different environments. The size distribution of cultivable airborne fungi was qualitatively the same regardless of the geographic region^4,5,27^ as it preserved a monomodal distribution with maximum concentrations in the fraction with an aerodynamic diameter of 2.1–3.3 μm (Figure 3). The dominant size for fungi was in the size range 2.1–4.7 μm, indicating their relatively higher size compared to bacteria.^1,5^ Previous studies also showed that airborne bacteria preserve bimodal size distributions, whereas fungal size distributions are unimodal.^1,27^ The typical size range of airborne microorganisms is between 1 and 5 μm.^50^

The dominant wind direction was the north (E, NE, N, NW, W) during the sampling period (19/10/2021–22/10/2021). The fungal size distribution exhibited the same profile regardless of air mass origin, yet small variations regarding the contribution of the coarse fraction (>7 μm) of the bacterial size distribution were observed.

### 3.2 Τοtal Airborne Bacteria (16S rRNA)

#### 3.2.1 Concentrations

The concentrations of total airborne bacteria identified by molecular techniques (qPCR) are given for each sampling day in Figure 4a. Bacterial concentrations varied between 44.4 and 923.7 GE/m^3^ with samples on 04/11 and 07/11 characterized by substantially higher concentrations. During these 2 days, intense Saharan dust transport events took place, thus higher concentrations are attributed to a significant number of transported microorganisms. In more detail, five out of the seven collected samples (4th to 8th of November) corresponded to days with aerosol origin from the south and above the Sahara desert as FLEXPART simulations indicated in Gao et al.^14^ Given that the bacteria sampled through this campaign had a dominant contribution from desert dust particles, elevated bacterial concentrations on dusty days have been observed in other locations.^51^

**Figure 4:**
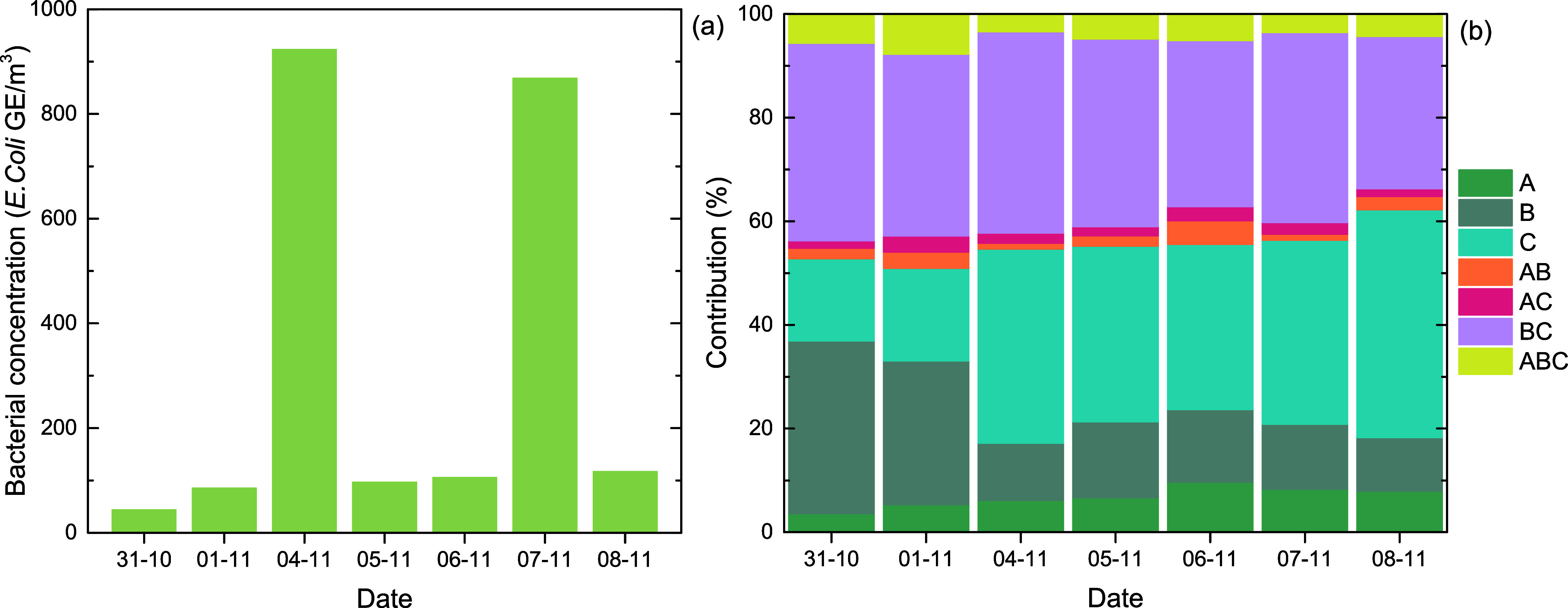
(a) Total airborne bacterial concentrations (16S rRNA) per sampling day and (b) % contribution of fluorescent particles in each WIBS channel for the same dates.

However, evaluation of bacterial concentrations at (HAC)^2^ indicates that concentrations at high altitude were considerably lower than those measured at ground-level samplings. Median bacterial concentration in this study was 105.8 GE/m^3^, whereas in a coastal site located in Crete (Akrotiri station, Chania), whereby the same analysis techniques were applied, median concentration was 2,110 GE/m^3^.^5^ Microorganism concentrations are reduced at high altitude due to harsher environmental conditions such as the lower temperature and exposure to UV light.^2^

Besides the impact from Sahara dust episodes on the measured quantities, (HAC)^2^ was inside the planetary boundary layer (PBL) during all (active) samplings.^13^ Therefore, airborne bacteria are associated with characteristics and sources of the surrounding biota of the study site rather than with the influence posed by air masses coming from the free troposphere.

#### 3.2.2 Relationships

Investigating the relationship between molecularly identified bacteria (16S rRNA) and fluorescent particles (WIBS) shows that statistically high correlations were obtained only with A_WIBS_ (0.82, *p* < 0.05) and C_WIBS_ (0.82, *p* < 0.05) channels (Table 2a). In both cases, there is a strong positive monotonic relationship. Figure 4b, which presents the % contribution for each WIBS channel, shows that particles in A_WIBS_ and C_WIBS_ are increased for the same dates, with particles at C_WIBS_ having higher contributions (16–44%) compared to particles at A_WIBS_ (4–10%). Savage et al.^52^ found that bacteria are dominated by fluorescent particles in channel A_WIBS_, while Gao et al.^14^ reported that fluorescent particles in channel C_WIBS_ are associated with mineral dust from the Sahara desert. The present findings verify previous observations, as experimental techniques in this study focused on the quantification of total airborne bacteria in conjunction with sampling campaigns on days that were significantly affected by dust transport. For the remaining channels, Spearman correlations were not statistically significant, with higher values obtained for AC_WIBS_ (0.43) and BC_WIBS_ (0.46) most likely due to the contribution of mineral dust through C_WIBS_ channel (Table 2a).

**Table 2:** Spearman Correlation between (a) Airborne Bacterial Concentrations (16S rRNA), Ice Nucleation Particles (INP), and WIBS Fluorescent Particles, (b) Airborne Bacterial Concentrations (16S rRNA), Ice Nucleation Particles (INP), and Particle Volume Concentration at Variable Size Classes (V1 = 1 μm, V2.5 = 1 μm etc), and (c) Airborne Bacterial Concentrations (16S rRNA), Ice Nucleation Particles (INP), and Organics and Ions (SO_4_^2–^, NH_4_^+^, NO_3_^–^, Cl^–^)­^a^

(a)	**A**_**WIBS**_	**B**_**WIBS**_	**C**_**WIBS**_	**AB**_**WIBS**_	**AC**_**WIBS**_	**BC**_**WIBS**_	**ABC**_**WIBS**_
bacteria (16S rRNA)	0.82*	–0.04	0.82*	–0.18	0.43	0.46	–0.04
INP	0.43	0.39	0.32	0.36	0.25	0.39	0.43

(b)	**V1**	**V2.5**	**V10**	**V1–10**	**INP**
bacteria (16S rRNA)	0.61	0.86*	0.82*	0.82*	–0.04
INP	0.61	0.25	0.21	0.21	

(c)	**Organics**	**SO**_**4**_^**2–**^	**NH**_**4**_^**+**^	**NO**_**3** ^**–**^_	**Cl** ^**–**^
bacteria (16S rRNA)	–0.71	–0.46	–0.85*	–0.85*	–0.61
INP	0.29	0.71	0.19	0.20	0.41

a The asterisk denotes statistically significant results (*p* < 0.05).

Especially, ABC_WIBS_ particles showed no relationship with airborne bacteria (−0.04, Table 2a) while a positive monotonic relationship with INP was found (0.43, Table 2a). Their % contribution on total fluorescent particles varied between 3% and 8% (Figure 4b), and a higher correlation was obtained with Cl^–^ (0.61). INP, on the other hand, had higher positive correlation with fine particles (V1; 0.61, Table 2b) and SO_4_^2–^ (0.71, Table 2c) and no correlation with airborne bacteria (−0.04, Table 2b). In addition, correlations with particle volume concentrations demonstrate that the measured bacteria had a significant partition in the supermicrometer size range, as statistically significant correlations were found for all size classes above 1 μm (Table 2c). These findings suggest that fluorescent particles identified as ABC at (HAC)^2^ during the present campaign cannot be exclusively associated with bacteria. Sampling time and the bioaerosol diurnal cycle can play a role in the observed characteristics. The type of fluorescent particles (e.g., A, B, AB, etc.) can be characteristically linked with the detection of biological particles of different types (e.g., pollen, fungi, bacteria). Savage et al.^52^ found that ABC particles are identified as several types of pollen and fungi. Inherently, fungi are found airborne in larger sizes compared to bacteria,^1^ also evidenced by the measurements of cultivable microorganisms at (HAC)^2^ (Section 3.1).

Likewise, INP may not be associated with airborne bacteria at (HAC)^2^ although it seems there is a possible link with ABC_WIBS_ particles and a link of higher certainty with fine particles.^14,25^ Wright et al.^53^ measured both INP and fluorescent aerosols and have associated INP presence with fungal spores, although no measurements of microorganisms were performed. Fragmented pollen has also been identified as type ABC, B, or BC fluorescent particles.^54^ In the absence of further evidence, INP at (HAC)^2^ could be associated with fragmented fungi or pollen. In any case, the present results indicate a successful detection of airborne bacteria by A_WIBS_ when (HAC)^2^ is in the PBL, thus near-ground biota is the dominant pool for bioaerosol.^14^ Contamination from interfering particles such as dust particles in this campaign can lead to uncertainties, yet the present findings underline that the choice of the type of fluorescent particles representing biological entities in a site should be done considering both the type of biological particles and their relevant size.

At the end, Spearman correlations between total bacterial (16S rRNA) and organics and ions (SO_4_^2–^, NH_4_^+^, NO_3_^–^, Cl^–^) showed negative relations with correlations ranging between −0.46 (SO_4_^2–^) and −0.85 (NH_4_^+^, NO_3_^–^; *p* < 0.05) (Table 2c). These negative correlations are translated into inverse relationships between the measured quantities. Most likely the reason behind the inverse relationships emerges from aerosol/bioaerosol sizes. Bacteria are known to lie predominantly in the micron size range (>1 μm)^55^ as also found in this study (Table 2b). Contrarily, the organics and ions measured at (HAC)^2^ had an upper threshold at 1 μm.^26^ In this way, partition at different sizes for the measured species can be translated to different contributing sources.

### 3.3 Taxonomic Diversity of Airborne Bacterial Communities

A total of 254,248 qualified paired-end reads with an average count per sample of 36,321 reads were obtained after sequencing and quality filtering. Based on a 97% species similarity, 123 bacterial OTUs classified in 10 phyla, 16 classes, 56 families, and 78 genera were obtained across all samples. Most of the bacteria identified belonged to phyla Actinobacteriota, Proteobacteria, and Firmicutes, with the following relative abundance (RA) values per sampling event: 9.7–66.3%, 12.8–68.7%, and 10.0–19.7%, respectively. The rest of the phyla, namely, Patescibacteria, Chloroflexi, Deinococcota, Bacteroidota, Thermotogota, Cyanobacteria, and Gemmatimonadota, accounted for 1.9–4.0% of each sampling event RA.

The most abundant bacterial classes were Actinobacteria (9.3–63.0%), Alphaproteobacteria (12.2–63.4%), Bacilli (9.0–18.1%), and Gammaproteobacteria (0.1–5.4%). These taxa together accounted for 90.9–96.2% of total abundance. The rest of the classes showed total RA < 10% per sampling event. The genera abundances of different samples showed high variation. The 12 most abundant bacterial genera are shown in Table 3, with their respective RA values (%). Their mean RA values during the whole sampling period accounted together for 66.4% of total abundance.

**Table 3:** Relative Abundance (%) of the 12 Most Abundant Airborne Bacterial Genera

	relative abundance (%)			
genus	mean	min	max	STD
Uncultured *Actinobacteria* genus	12.1	0.2	26.3	9.7
*Geodermatophilus*	10.3	0	19.7	8
*Allorhizobium-Neorhizobium-Pararhizobium-Rhizobium*	9.8	0	50.1	18.6
*Corynebacterium*	7.4	0	47	17.1
*Bacillus*	6.2	0.8	13.4	4.6
*Cellulomonas*	4.3	0	7.2	3.1
*Kocuria*	3.9	0.1	10.7	3.5
*Pseudarthrobacter*	3.8	0.7	5.7	1.8
*Rubellimicrobium*	3.3	0	8.3	3.3
*Blastococcus*	3.1	0.5	6.1	2.1
*Staphylococcus*	2.2	0	11.1	4

Similarities and differences were observed in the bacterial composition across different sampling dates (Figure 5). On 31st October, the bacterial community was dominated by the group *Allorhizobium-Neorhizobium-Pararhizobium-Rhizobium* (50.1%), followed by *Staphylococcus* and *Corynebacterium* (11.1% and 3.3%, respectively). On the 1st of November, a significant increase in *Corynebacterium* was observed (46.1%), which dominates the bacterial community, while the RA of the group *Allorhizobium-Neorhizobium-Pararhizobium-Rhizobium* decreased to 15.4%. From the 4th of November, a slight difference was observed in bacterial composition across the sampling dates, with a prevalence of members of Actinobacteria phylum including *uncultured_Actinobacteria* (RA: 12.8%–23.6%), *Geodermatophilus* (RA: 11.7%–18.3%) *Cellulomonas* (RA: 3.6%–7.6%), and *Kocuria* (RA: 3.4%–6.6%). The RA of the *Bacillus* genus increased from the 4th of November with a slight variation between the sampling dates (RA: 6.7%–11.2%). *Rubellimicrobium* showed a slight increase mainly on the 4th and 7th of November (7.1% and 7.9%, respectively).

**Figure 5:**
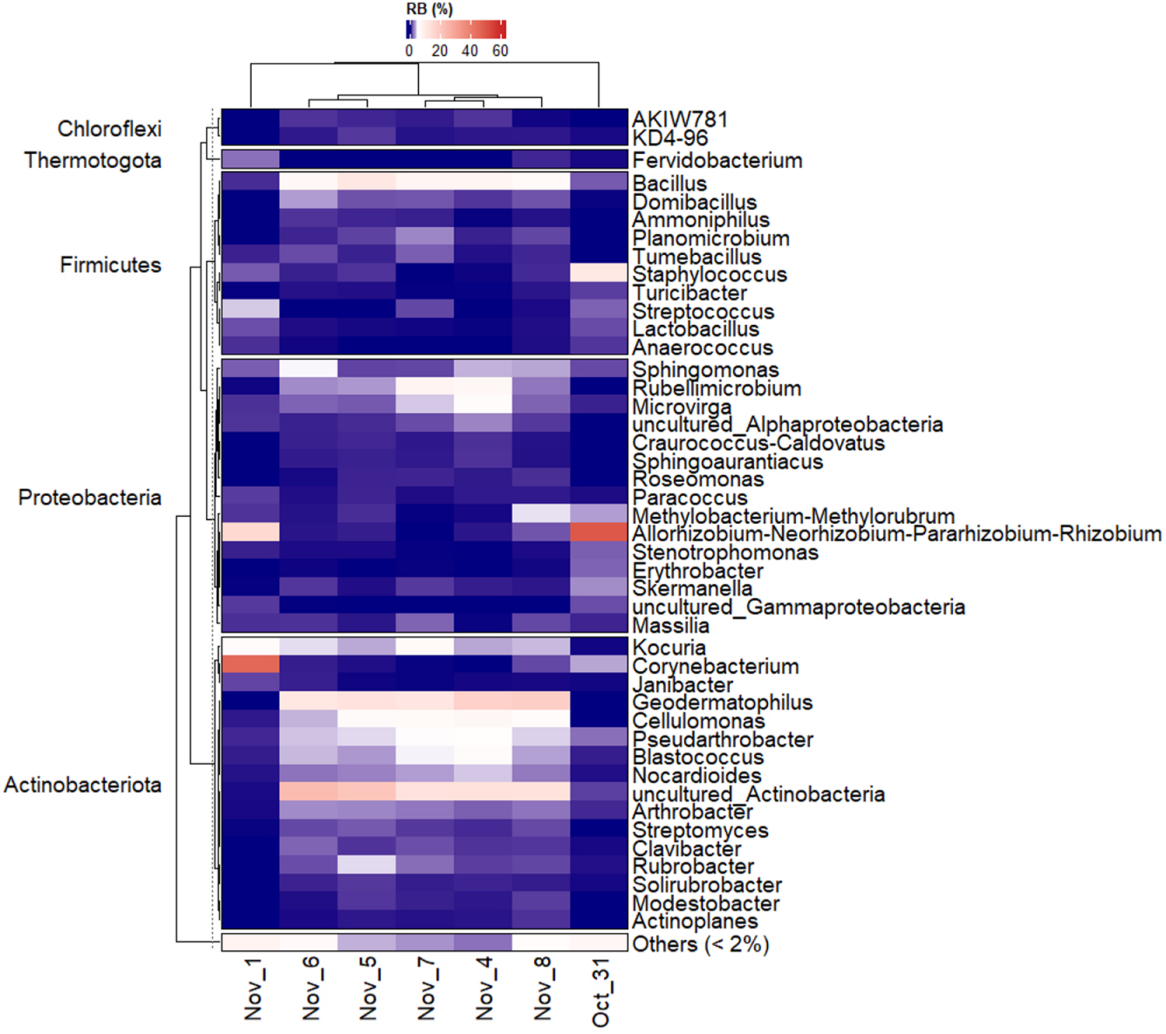
Relative abundances (RA) of identified bacterial genera are grouped by phyla. Data were agglomerated to genus level and mean RA values per sampling day were calculated. Only genera that accounted for at least 2% of total RA are indicated.

Most ice nucleation active bacteria were identified as members of known and unknown bacterial species in the *Pseudomonadaceae*, *Enterobacteriaceae*, and *Xanthomonadaceae* families, along with members of the *Lysinibacillus* genus of the *Planococcaceae* family.^56,57^ In this study, a minor community of *Klebsiella* and *Stenotrophomonas* members of *Enterobacteriaceae* and *Xanthomonadaceae*, respectively, were identified mainly on 31st October and 1st November, constituting 0.3% to 1.9% of the bacterial community, while *Lysinibacillus* genus member of *Planococcaceae* was detected after the 4th of November, with a relative abundance below 1% (Figure 6). Even though a number of ice nucleation bacteria were identified in the field study, no specific correlation was found with INP measurements. It is likely that the relative abundances of these species did not significantly influence the *in situ* ice nucleation.

**Figure 6:**
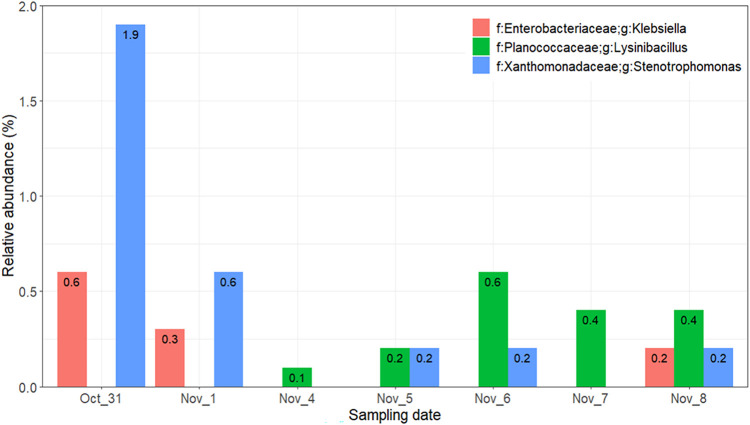
Relative abundance of the identified potential ice nucleation active bacteria across the sampling dates.

### 3.4 Influence of Dust Episodes on Microorganism Abundance

#### 3.4.1 Analysis of Back Trajectories Using the FLEXPART Model

The bacterial genera abundance during the sampling period showed high variability in relation to the origin of the air masses origin. Between November 4th and November 8th, a southern direction was dominant with air masses originating predominantly from the Saharan region^14^ (Figure 7). The residence time in each geographic grid cell (1° × 1 °) corresponded to a height up to 500 m a.g.l. in order to investigate the origin of air masses within the boundary layer. Accordingly, on the 31st of October and the 1st of November, air masses originated mainly from the northeast, while on the 4th–8th of November, air masses originated from the south–southwest.

**Figure 7:**
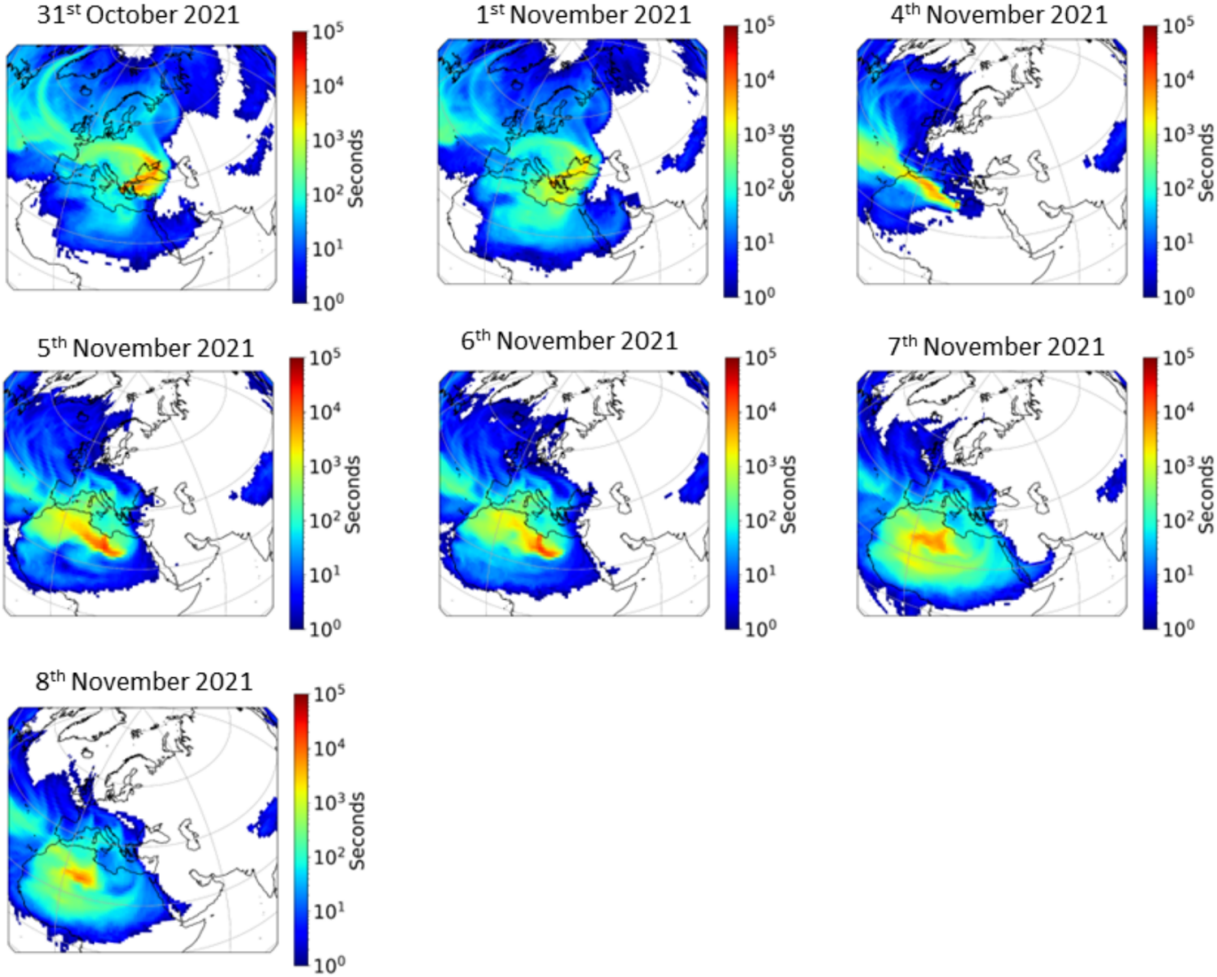
Flexpart simulations of the air masses arriving over the Helmos (HAC)^2^ site (31 October to 8 November 2021).

#### 3.4.2 Analysis of Air Mass Origin Using Lidar Measurements

The spatiotemporal evolution of the range-corrected lidar signals at 1064 nm, between 1.8 and 6 km a.s.l., is presented in Figure 8, as obtained by AIAS from 4th to 8th of November 2021. In more detail, on November 4th (Figure 8a), a descending thin aerosol layer was observed between 3 and 3.5 km (15:00 UTC) reaching the (HAC)^2^ around 18:00 UTC. Subsequently, during morning hours on November 5th (10:00–16:00 UTC), three intense dust layers were observed between 2.2 and 4.5 km where it is seen that the lowest layer (brown color) brings high aerosol loads at (HAC)^2^ height.

**Figure 8:**
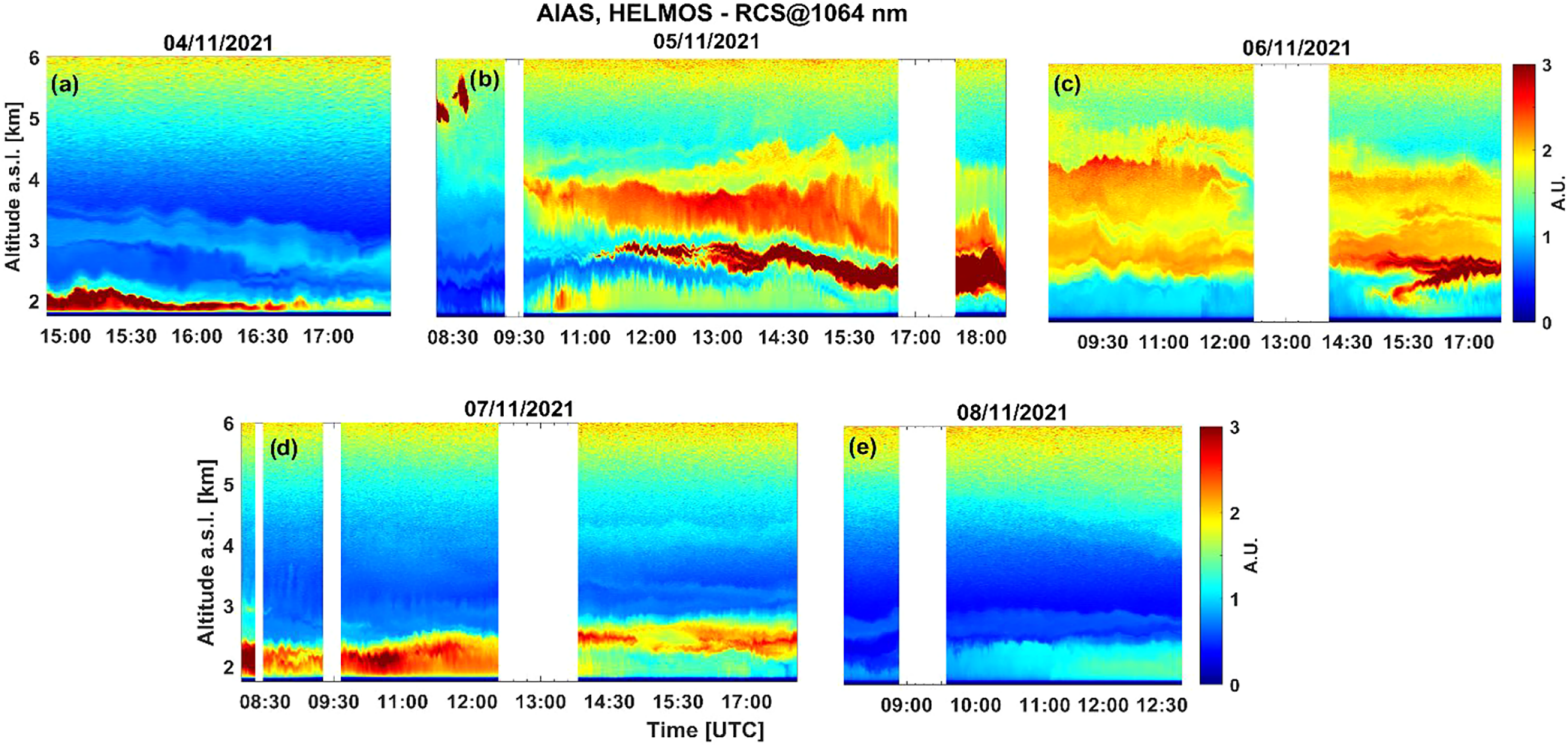
Spatiotemporal evolution of the range-corrected signal (RCS) lidar signal retrieved by the AIAS lidar at 1064 nm in arbitrary units (A.U.) over (HAC)^2^ (4th to 8th November 2021) up to 6.0 km height a.s.l.

Moreover, on November 5th (Figure 8b, 17:00–18:00 UTC), a thick aerosol layer (dark brown color) was located at (HAC)^2^ height between 2.2 and 2.8 km. On November 6th (Figure 8c), two distinct filamented aerosol layers were observed (2.5–3.0 and 3.5–4.4 km) from 08:30 to 12:30 UTC. Around 14:00 UTC, the lower layer was intensified after 15:00 UTC around 2.5 km height, while a rising plume containing locally emitted aerosols appeared at altitudes between 2 and 2.5 km height. The AIAS lidar measurements on November 7th (Figure 8d) delineate a shallow and intense aerosol layer, from the ground up to 2.5 km height, in which (HAC)^2^ is continuously engaged in (08:24–12:28 UTC). In addition, heating of the ground led the air masses from the PBL to reach the higher altitudes, forming an intense aerosol layer between 2.2 and 2.6 km (14:00–18:00 UTC). Finally, on November 8th (Figure 8e) during early morning hours (08:15–09:15 UTC), (HAC)^2^ remains mainly in a near aerosol-free region, between two aerosol layers, one located above (2.5–2.8 km height) and another below (from ground up to 2.1 km height). Later on that day (09:50–12:45 UTC), the aerosol content in the lower atmosphere (from ground up to 2.5 km) increases sharply, indicating the arrival of PBL air masses originating from local sources.^13^

The above trajectory analysis and lidar measurements agree with results from the molecular analysis, which identified the presence of the species with relative abundance close to 7.6 ± 5.7% for the period 4–8/11/2021 (Figure 9a). During Saharan dust episodes, airborne bacteria at (HAC)^2^ were enriched mainly with the species of *Geodermatophilus africanus* and among other bacteria (*Cellulomonas* uncultured bacterium, , , *Bacillus* uncultured bacterium, *Blastococcus* uncultured bacterium, and *Microvirga* uncultured bacterium). is a Gram-positive, aerobic, and halotolerant bacterium from the genus *Geodermatophilus* which has been isolated from desert sand near Ourba in the Sahara and has been detected in dust events in Italy and Eastern Mediterranean.^58^ Moreover, *Actinomyces* was also found in some dust storm microbiology studies.^59^ It is noteworthy that the Gram-positive *Actinomyces* is listed in the World Health Organization’s^60^ global priority pathogens list of multidrug- and antibiotic-resistant bacteria.

**Figure 9:**
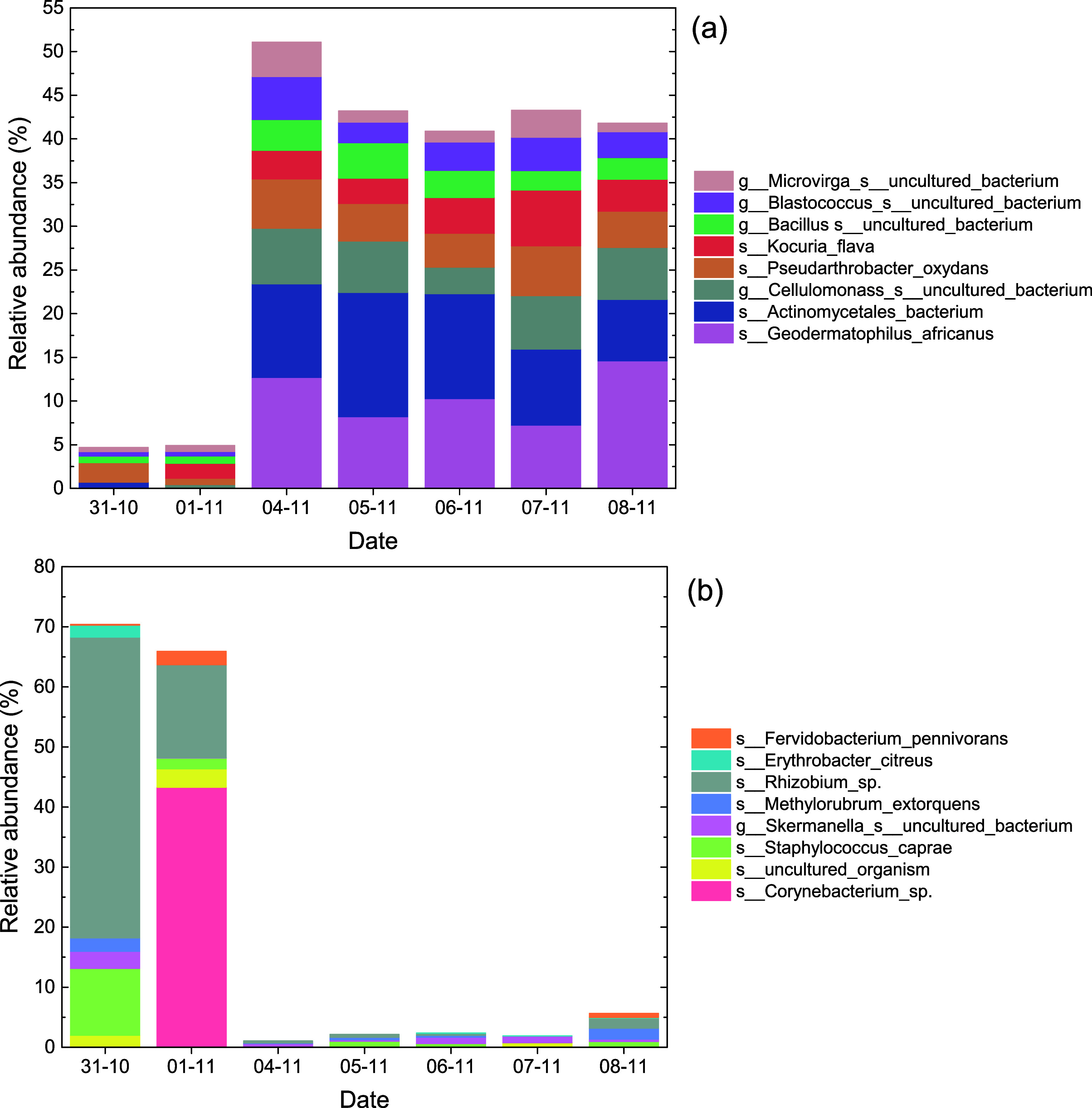
Relative abundance (%) of bacterial species (a) on Saharan dust episodes and (b) during continental air masses.

Contrarily, the abundance of the and was zero during the period 31/10–1/11/2021, where northern air masses were dominant.^14^ During continental air masses transport, species such as *Rhizobium sp*., *Corynebacterium sp*., and had a high relative abundance (Figure 9b).

## 4 Conclusions

The airborne microbial community was investigated at the free tropospheric background (HAC)^2^ site. Regarding the viable, cultivable, airborne microbes, the mean concentration of the potentially pathogenic, heterotrophic bacteria was exceptionally low, equal to 4 ± 4 CFU/m^3^ (value range 0–12 CFU/m^3^). In accordance with the culture-dependent analysis, relatively low total bacterial concentrations were also determined by real-time PCR, ranging from 33.4 to 117.2 GE/m^3^. Compared to the bacteria, fast-growing fungi were shown to be less affected by the low temperatures prevailing at the Helmos (HAC)^2^ site. Their average concentration was 182 ± 86 CFU/m^3^ (value range 72–314 CFU/m^3^). The particle size distribution of heterotrophic bacteria and fungi was similar to measurements performed close to the sea level. Bacterial size distributions were bimodal with one mode at fine sizes (1.1–2.1 μm) and a second mode at coarse sizes (>7 μm). On the other hand, the particle size distribution of the airborne fungi was monomodal with a dominant mode at 2.1–3.3 μm size fraction.

A good correlation was observed between the bacterial load in air and the online measurements of fluorescent particles, whereas no significant correlation was detected with the INP and chemical composition of particulate matter. Higher correlations were found with channels A_WIBS_ and C_WIBS_, suggesting the influence of near-ground biota in the first case and dust particles contributions in the second case. Surprisingly, although the total bacterial concentration quantified using real-time PCR was relatively low, high bacterial richness and species diversity of the total airborne bacterial community was encountered at the free tropospheric background (HAC)^2^ site (123 bacterial OTUs classified in 10 phyla, 16 classes, 56 families, and 78 genera). The bacterial community in each sampling day was influenced by the direction and origin of the air masses. During the Saharan dust episodes, there was an enrichment of specific species such as and , among others, evidencing their transport to (HAC)^2^ from southern regions. However, during the presence of continental air masses, species such as *Rhizobium sp*., *Corynebacterium sp*., and had a high relative abundance. Most importantly, the present results comprise a first proof of the varying contributions in bacterial populations and diversity events during short-term samplings in high-altitude sites such as (HAC)^2^.

## References

[1] Zhai Y., Li X., Wang T., Wang B., Li C., Zeng G. (2018). A review on
airborne microorganisms in particulate matters: Composition, characteristics
and influence factors. Environ. Int..

[2] Ariya P. A., Sun J., Eltouny N. A., Hudson E. D., Hayes C. T., Kos G. (2009). Physical and
chemical characterization of bioaerosols – Implications for
nucleation processes. Int. Rev. Phys. Chem..

[3] Després V. R., Huffman J. A., Burrows S. M., Hoose C., Safatov A. S., Buryak G. (2022). Primary
biological aerosol particles in the atmosphere:
A review. Tellus B.

[4] Katsivela E., Latos E., Raisi L., Aleksandropoulou V., Lazaridis M. (2017). Particle size distribution of cultivable
airborne microbes
and inhalable particulate matter in a wastewater treatment plant facility. Aerobiologia.

[5] Chatoutsidou S. E., Saridaki A., Raisi L., Katsivela E., Stathopoulou P., Tsiamis G., Voulgarakis A., Lazaridis M. (2023). Variations, seasonal shifts and ambient conditions
affecting airborne microorganisms and particles at a southeastern
Mediterranean site. Sci. Total Environ..

[6] Huang Z., Yu X., Liu Q., Maki T., Alam K., Wang Y., Xue F., Tang S., Du P., Dong Q., Wang D., Huang J. (2024). Bioaerosols in the atmosphere: A comprehensive review on detection
methods, concentration and infiltration factors. Sci. Total Environ..

[7] Knopf D. A., Alpert P. A. (2023). Atmospheric ice
nucleation. Nat.
Rev. Phys..

[8] DeLeon-Rodriguez N., Lathem T. L., Rodriguez L. M., Barazesh J. M., Anderson B. E., Beyersdorf A. J., Ziemba L. D., Bergin M., Nenes A., Konstantinidis K. T. (2013). The microbiome of the upper troposphere: species composition
and prevalence, effects of tropical storms, and atmospheric implications. Proc. Natl. Acad. Sci. U.S.A..

[9] Fröhlich-Nowoisky J., Weber C. J., Huffman B., Pohlker J. A., Andreae C., Lang-Yona M. O., Burrows N., Gunthe S. M., Elbert S. S., Su W., Hoor H., Thines P., Hoffmann E., Despers T., R V., Poschl U. (2016). Bioaerosols in the earth system: Climate, health and
ecosystem interactions. Atmos. Res..

[10] Teruya M., Kentaro H., Kevin C. L., Yasuhiro K., Mizuo K., Maoto U., Kazuyuki K., Yasuhito I. (2023). Vertical distribution
of airborne microorganisms over forest environments: A potential source
of ice-nucleating bioaerosols. Atmos. Environ..

[11] Georgakaki P., Billault-Roux A.-C., Foskinis R., Gao K., Sotiropoulou G., Gini M., Takahama S., Eleftheriadis K., Papayannis A., Berne A., Nenes A. (2024). Unraveling ice multiplication
in winter orographic clouds viain-situ observations, remote sensing
and modeling. npj Clim. Atmos. Sci..

[12] Lazaridis M. (2022). Study of the
immersion freezing theory using the classical nucleation framework. Atmosphere.

[13] Foskinis R., Gao K., Gini M. I., Diapouli E., Vratolis S., Granakis K., Zografou O., Kokkalis P., Komppula M., Vakkari V., Eleftheriadis K., Nenes A., Papayannis A. (2024). The Influence
of the Planetary Boundary Layer on the Atmospheric State at an Orographic
Site at the Eastern Mediterranean. Tellus B.

[14] Gao K., Vogel F., Foskinis R., Vratolis S., Gini I. M., Granakis K., Billault-Roux A.-C., Georgakaki P., Zografou O., Fetfatzis P., Berne A., Papagiannis A., Eleftheriadis K., Möhler O., Nenes A. (2024). (2024). Biological
and dust aerosol as sources of ice nucleating particles in the Eastern
Mediterranean: source apportionment, atmospheric processing and parameterization. Atmos. Chem. Phys..

[15] Chatziparaschos, M. ; Myriokefalitakis, S. ; Kalivitis, N. ; Daskalakis, N. ; Nenes, A. ; Gonçalves Ageitos, M. ; Costa-Surós, M. ; Pérez García-Pando, C. ; Vrekoussis, M. ; Kanakidou, M. Assessing the global contribution of marine, terrestrial bioaerosols, and desert dust to ice-nucleating particle concentrations EGUsphere 2024 10.5194/egusphere-2024-952, 2024.

[16] Seinfeld J. H., Bretherton C., Carslaw K. S., Coe H., DeMott P. J., Dunlea E. J. (2016). Improving our fundamental understanding of
the role of aerosol–cloud interactions in the climate system. Proc. Natl. Acad. Sci. U.S.A..

[17] Sands D. C., Langhans V. E., Scharen A. L., de Smet G. (1982). The association
between
bac-teria and rain and possible resultant meteorological implications. J. Hung. Meteorol. Serv..

[18] Bauer H., Giebl H., Hitzenberger R., Kasper-Giebl A., Reischl G., Zibuschka F., Puxbaum H. (2003). Airborne bacteria as
cloud condensation nuclei. J. Geophys. Res..

[19] Sharma P. K., Rao K. H. (2002). Analysis of different
approaches for evaluation of
surface energy of microbial cells by contact angle goniometry. Adv. Colloid Interface Sci..

[20] Hartmann S., Augustin S., Clauss T., Wex T., Santl-Temkiv T., Voigtlander H., Niedermeier D., Stratmann F. (2013). Immersion
freezing of ice nucleation active protein complexes. Atmos. Chem. Phys..

[21] Prisle N. L., Lin J. J., Purdue S., Lin H., Meredith J. C., Nenes A. (2019). CCN activity of six pollenkitts and
the influence of their surface
activity. Atmos. Chem. Phys..

[22] Lazaridis M. (2019). A theoretical
study on the activation of insoluble particles in atmospheric conditions. Atmos. Res..

[23] Burrows S. M., Elbert W., Lawrence M. G., Poschl U. (2009). Bacteria in the global
atmosphere – Part 1: Review and synthesis of literature data
for different ecosystems. Atmos. Chem. Phys..

[24] Coen M. C., Andrews E., Aliaga D., Andrade M., Angelov H., Bukowiecki N., Ealo M., Fialho P., Flentje H., Hallar A. G., Hooda R., Kalapov I., Krejci R., Lin N.-H., Marinoni A., Ming J., Nguyen N. A., Pandolfi M., Pont V., Ries L., Rodríguez S., Schauer G., Sellegri K., Sharma S., Junying S., Tunved P., Velasquez P., Ruffieux D. (2018). (2018). Identification
of topographic influencing aerosol observations at high altitude stations. Atmos. Chem. Phys..

[25] Gao, K. ; Vogel, F. ; Foskinis, R. ; Vratolis, S. ; Gini, M. ; Granakis, K. ; Zografou, O. ; Fefatzis, P. ; Papagiannis, A. ; Möhler, O. ; Eleftheridadis, K. ; Nenes, A. Diurnal cycle of bioaerosols is a key driver of ice nucleating particle variability for Eastern Mediterranean orographic clouds npj Atmos.Climate Sci. 2025. 10.1038/s41612-024-00817-9 PMC12052592 40337146

[26] Zografou O., Gini M., Fetfatzis P., Granakis K., Foskinis R., Manousakas M. I., Tsopelas F., Diapouli E., Dovrou E., Vasilakopoulou C. N., Papayannis A., Pandis S. N., Nenes A., Eleftheriadis K. (2024). (2024). High-altitude
aerosol chemical characterization
and source identification: insights from the CALISHTO campaign. Atmos. Chem. Phys..

[27] Raisi L., Aleksandropoulou V., Lazaridis M., Katsivela E. (2013). Size distribution
of viable, cultivable, airborne microbes and their relationship to
particulate matter concentrations and meteorological conditions in
a Mediterranean site. Aerobiologia.

[28] Nadkarni M. A., Martin F. E., Jacques N. A., Hunter N. (2002). Determination
of bacterial
load by real-time PCR using a broad-range (universal) probe and primers
set. Microbiology.

[29] Chatoutsidou S. E., Saridaki A., Raisi L. L., Katsivela E., Tsiamis G., Zografakis M., Lazaridis M. (2021). Airborne particles
and microorganisms in a dental clinic: Variability of indoor concentrations,
impact of dental procedures, and personal exposure during everyday
practice. Indoor Air.

[30] Saridaki A., Glytsos T., Raisi L., Katsivela E., Tsiamis G., Kalogerakis N., Lazaridis M. (2023). Airborne particles,
bacterial and fungal communities insights of two museum exhibition
halls with diverse air quality characteristics. Aerobiologia.

[31] Ruijter J. M., Villalba A. R., Hellemans J., Untergasser A., van den Hoff M. J. B. (2015). Removal of between-run variation
in a multi-plate qPCR
experiment. Biomol. Detect. Quantif..

[32] Klindworth A., Pruesse E., Schweer T., Peplies J., Quast C., Horn M., Glöckner F. O. (2013). Evaluation
of general 16S ribosomal
RNA gene PCR primers for classical and next-generation sequencing-based
diversity studies. Nucleic Acids Res..

[33] Edgar R. C. (2010). Search
and clustering orders of magnitude faster than BLAST. Bioinformatic.

[34] Bolyen E., Rideout J. R., Dillon M. R. (2019). (2019).
Reproducible,
interactive, scalable, and extensible microbiome data science using
QIIME 2. Nat. Biotechnol..

[35] Edgar R. C. (2013). UPARSE:
highly accurate OTU sequences from microbial amplicon reads. Nat. Methods.

[36] Camacho C., Coulouris G., Avagyan V., Ma N., Papadopoulos J., Bealer K., Madden T. L. (2009). BLAST+: architecture and applications. BMC Bioinf..

[37] Quast C., Pruesse E., Yilmaz P., Gerken J., Schweer T., Yarza P. (2012). The SILVA ribosomal
RNA gene database project: improved
data processing and web-based tools. Nucleic
Acids Res..

[38] Möhler O., Adams M., Lacher L., Vogel F., Nadolny J., Ullrich R., Boffo C., Pfeuffer T., Hobl A., Weiß M., Vepuri H. S. K., Hiranuma N., Murray B. J. (2021). The Portable
Ice Nucleation Experiment (PINE): A New Online Instrument for Laboratory
Studies and Automated Long-Term Field Observations of Ice-Nucleating
Particles. Atmos. Meas. Tech..

[39] Fröhlich R., Cubison M. J., Slowik J. G., Bukowiecki N., Canonaco F., Croteau P. L., Gysel M., Henne S., Herrmann E., Jayne T., Steinbacher M., Worsnop D. R., Baltensperger U., Prévôt A. S. H. (2015). Fourteen
months of on-line measurements of the non-refractory submicron aerosol
at the Jungfraujoch (3580 m a.s.l.) – chemical composition,
origins and organic aerosol sources. Atmos.
Chem. Phys..

[40] Ogren J. A., Wendell J., Andrews E., Sheridan P. J. (2017). Continuous light
absorption photometer for long-term studies. Atmos. Meas. Technol..

[41] Wiedensohler A., Birmili W., Nowak A., Sonntag A., Weinhold K., Merkel M., Wehner B., Tuch T., Pfeifer S., Fiebig M., Fjäraa A. M., Asmi E., Sellegri K., Depuy R., Venzac H., Villani P., Laj P., Aalto P., Ogren J. A., Swietlicki E., Williams P., Roldin P., Quincey P., Hüglin C., Fierz-Schmidhauser R., Gysel M., Weingartner E., Riccobono F., Santos S., Grüning C., Faloon K., Beddows D., Harrison R., Monahan C., Jennings S. G., O’Dowd C. D., Marinoni A., Horn H.-G., Keck L., Jiang J., Scheckman J., McMurry P. H., Deng Z., Zhao C. S., Moerman M., Henzing B., de Leeuw G., Löschau G., Bastian S. (2012). Mobility particle size spectrometers: harmonization
of technical standards and data structure to facilitate high quality
long-term observations of atmospheric particle number size distributions. Atmos. Meas. Technol..

[42] Papayannis A., Kokkalis P., Mylonaki M., Soupiona R., Papanikolaou C. A., Foskinis R., Giakoumaki A. (2020). Recent upgrades
of the EOLE andAIAS
lidar systems of the National Technical University of Athens operating
since 2000 in Athens, Greece. EPJ Web Conf..

[43] Mylonaki M., Papayannis A., Papanikolaou C.-A., Foskinis R., Soupiona O., Maroufidis G., Anagnou D., Kralli E. (2021). Tropospheric vertical
profiling of the aerosol backscatter coefficient and the particle
linear depolarization ratio for different aerosol mixtures during
the PANACEA campaign in July 2019 at Volos, Greece. Atmos. Environ..

[44] Stohl A., Forster C., Frank A., Seibert P., Wotawa G. (2005). Technical
note: The Lagrangian particle dispersion model FLEXPART version 6.2. Atmos. Chem. Phys..

[45] Pisso I., Sollum E., Grythe H., Kristiansen N. I., Cassiani M., Eckhardt S., Arnold D., Morton D., Thompson R. L., Groot Zwaaftink C. D., Evangeliou N., Sodemann H., Haimberger L., Henne S., Brunner D., Burkhart J. F., Fouilloux A., Brioude J., Philipp A., Seibert P., Stohl A. (2019). The Lagrangian particle dispersion
model FLEXPART version 10.4. Geosci. Model Dev..

[50] Wei M., Xu C., Xu X., Zhu C., Li J., Lv G. (2019). (2019). Size
distribution of bioaerosols from biomass burning emissions: characteristics
of bacterial and fungal communities in submicron (PM1.0) and fine
(PM2.5) particles. Ecotoxicol. Environ. Saf..

[51] Amarloei A., Fazlzadech M., Jafari A. J., Zarei A., Mazloomi S. (2020). Particulate
matters and bioaerosols during Middle East dust storms events in Ilam,
Iran. Microchem. J..

[52] Savage N. J., Krentz C. E., Könemann T., Taewon T. H., Mainelis G., Pöhlker C., Huffman J. A. (2017). (2017). Systematic characterization
and fluorescence threshold strategies for the wideband integrated
bioaerosol sensor (WIBS) using size-resolved biological and interfering
particles. Atmos. Meas. Technol..

[53] Wright T. P., Hader J. D., McMeeking G. R., Petters M. D. (2014). High Relative Humidity
as a Trigger for Widespread Release of Ice Nuclei. Aerosol Sci. Technol..

[54] Hughes D. D., Mampage C. B. A., Jones L. M., Liu Z., Stone E. A. (2020). Characterization
of Atmospheric Pollen Fragments during Springtime Thunderstorms. Environ. Sci. Technol. Lett..

[55] Li Y., Lu R., Li W., Xie Z., Song Y. (2017). Concentrations and
size distributions of viable bioaerosols under various weather conditions
in a typical semi-arid city of Northwest China. J. Aerosol. Sci..

[56] Failor K. C., Schmale D. G., Vinatzer B. A., Monteil C. L. (2017). Ice Nucleation Active
Bacteria in Precipitation Are Genetically Diverse and Nucleate Ice
by Employing Different Mechanisms. ISME J..

[57] Lukas M., Schwidetzky R., Eufemio R. J., Bonn M., Meister K. (2022). Toward Understanding
Bacterial Ice Nucleation. J. Phys. Chem. B.

[58] Behzad H., Katsuhiko M., Takashi G. (2018). Global Ramifications
of Dust and
Sandstorm Microbiota. Genome Biol. Evol..

[59] Griffin D. W., Westphal D. L., Gray M. A. (2006). Airborne
microorganisms in the African
desert dust corridor over the mid-Atlantic ridge, Ocean Drilling Program,
leg 209. Aerobiologia.

[60] World Health Organization . Prioritization of Pathogens to Guide Discovery, Research and Development of New Antibiotics for Drug-Resistant Bacterial Infections Including Tuberculosis; World Health Organization: Geneva, 2017.

